# Blocking mineralocorticoid signaling with esaxerenone reduces atherosclerosis in hyperglycemic ApoE KO mice without affecting blood pressure and glycolipid metabolism

**DOI:** 10.1038/s41598-025-95324-z

**Published:** 2025-03-29

**Authors:** Hideyuki Iwamoto, Junpei Sanada, Tomohiko Kimura, Masashi Shimoda, Yuichiro Iwamoto, Kazunori Dan, Yoshiro Fushimi, Yukino Katakura, Yuka Nogami, Yoshiko Shirakiya, Yuki Yamasaki, Shuhei Nakanishi, Tomoatsu Mune, Kohei Kaku, Hideaki Kaneto

**Affiliations:** Department of Diabetes, Endocrinology and Metabolism, Kawasaki Medical School, 577 Matsushima, Kurashiki, 701-0192 Japan

**Keywords:** Esaxerenone, Oxidative stress, Atherosclerosis, Cardiology, Endocrinology

## Abstract

Endothelial damage mediated by mineralocorticoid receptor (MR) is an important factor in the development of atherosclerosis. Esaxerenone is a highly selective drug that can specifically block MR activity. The aim of this study is to examine whether specific blocking of mineralocorticoid signaling with esaxerenone exerts favorable effects on the progression of atherosclerosis. ApoE KO mice were used as a model of atherosclerosis. In addition to a non-diabetic model, we created a diabetic model using streptozotocin. These were divided into a control group and an esaxerenone group. Esaxerenone-containing diet was provided for 8 weeks starting at 10 weeks of age. Various metabolic markers and abdominal aortic mRNA expression were evaluated, and histological examination of the aortic arch and thoracic aorta was performed. We also used human aortic smooth muscle cells (HASMCs) to investigate the possible direct effects of esaxerenone on vascular smooth muscle cells. In diabetic mice, plaque area in the aortic arch was significantly smaller in esaxerenone group compared to control group, although there were no differences in blood pressure, serum lipid levels between the two groups. Inflammation-related genes, macrophage marker, cell adhesion factors and oxidative stress marker were all significantly lower in esaxerenone group. The studies using HASMCs have confirmed that esaxerenone has anti-inflammatory effects on vascular smooth muscle cells. Specific blocking of mineralocorticoid signaling with esaxerenone exerts favorable effects on the progression of atherosclerosis without influencing blood pressure and glycolipid metabolism.

**Supplementary Information:**

The online version contains supplementary material available at 10.1038/s41598-025-95324-z.

## Introduction

Atherosclerotic cardiovascular disease is a chronic inflammatory disease of intima of the aorta and medium-sized arteries, and it accounts for one third of all deaths worldwide^1^. An important risk factor for atherosclerosis is the dysfunction of vascular endothelial cells due to chronic inflammation, which is often observed in subjects with metabolic syndrome or diabetes mellitus^2–9^. Chronic inflammation is mainly caused by the infiltration and accumulation of inflammatory cells such as macrophages and lymphocytes, and these cells release inflammatory cytokines^10–14^. On the other hand, it has been reported that the renin-angiotensin system (RAS) is activated in blood vessels with atherosclerosis, and mineralocorticoid receptor (MR)-mediated vascular endothelial dysfunction also contributes to the development of atherosclerosis^15^. In subjects with obesity, hyperglycemia and excessive salt intake, aldosterone activates MR signaling, which finally provokes oxidative stress through the activation of nicotinamide adenine dinucleotide phosphate (NADPH) oxidase. MR is also expressed in vascular endothelial cells, vascular smooth muscle cells and macrophages^16^, and it has been thought that its activation leads to the progression of atherosclerosis independently of hypertension. It has been also reported that primary aldosteronism increases the incidence of cardiovascular disease independently of blood pressure compared to essential hypertension^17^.

Therefore, we hypothesized that the progression of atherosclerosis could be inhibited by suppressing MR activity using an MR blocker. In this study, we blocked mineralocorticoid signaling with esaxerenone, an MR blocker that is widely used in clinical practice. There are several reports on basic research using esaxerenone. For example, it has been reported that esaxerenone shows renoprotective effect independently of blood pressure in type 2 diabetes model mice and cardioprotective effect using hypertension model rats^18,19^. In the FIDELIO-DKD study, the risk of developing a composite renal endpoint was significantly reduced by 18% with finerenone, a non-steroidal MR antagonist, which was similar to esaxerenone^20^. However, there has been no report at all showing the possible effects of MR blocking on the progression of atherosclerosis. The purpose of this study was to examine whether blocking of mineralocorticoid signaling with esaxerenone exerts anti-atherosclerotic effects and to elucidate the molecular mechanisms of the possible anti-atherosclerotic effects of blocking mineralocorticoid signaling under diabetic and non-diabetic conditions.

## Methods

### Animals and diets

ApoE knockout mice (C57BL/6 J-ApoEtm1Unc) were purchased from Charles River Laboratories and reared under controlled environmental conditions on a 12-h light/dark cycle (2 mice per cage in all experiments). Mice were fed water and standard diet (MF; Oriental Yeast Co.) were fed ad libitum until 8 weeks of age, and the room temperature was maintained at 25 °C.

We set diabetes model and non-diabetes model. For making diabetes model, at 8 weeks of age, we used streptozotocin (STZ) to induce hyperglycemia based on our previous report^21^. Mice were injected intraperitoneally with STZ (50 mg/kg/day) (Fujifilm Wako Pure Chemicals) for 5 consecutive days. Mice that showed obvious hyperglycemia of 300 mg/dL or more under feeding conditions were designated as a diabetic model. At the age of 10 weeks, the diabetic and non-diabetic models were respectively divided into a standard diet group and an esaxerenone (Provided by Daiichi Sankyo Co., LTD.) -containing diet group (3 mg/kg/day) for intervention. With regard to the dosage of esaxerenone, we referred to the previous report^22^, and administered esaxerenone mixed with normal food at a concentration of 0.003% based on the average amount of food intake. Mice in the four groups were observed from 10 to 18 weeks of age, and body weight, blood glucose, and food intake were measured throughout the experimental period. In addition, we also investigated the effects of spironolactone. Spironolactone (FUJIFILM Wako Pure Chemical Corporation) was suspended in a 0.5% sodium carboxymethylcellulose solution and, as with esaxerenone, was administered orally (50 mg/kg/day) to ApoE-deficient male diabetic model mice and normal blood glucose model mice from 10 to 18 weeks of age^23,24^.

This study was approved by the Animal Use Committee of Kawasaki Medical School (No. 23–037) and was conducted in accordance with the Kawasaki Medical School Animal Use Guidelines. After the experiments, the mice were killed by cervical dislocation under inhalation anesthesia with sevoflurane. This experimental procedure was performed according to the ARRIVE guidelines.

### Oral glucose tolerance test (OGTT)

After 16 h fasting, animals were given d-(+)-glucose (1 g/kg BW) was administered orally for OGTT at 10 weeks old. We performed OGTT again after 8-week treatment with spironolactone and esaxerenone. Blood samples were collected at 0, 15, 30, 60, 90 and 120 min after glucose load and blood glucose levels were measured using Glutest Mint. Serum insulin levels were determined using a mouse insulin ELISA kit (Morinaga, Tokyo, Japan).

### Insulin tolerance test (ITT)

Insulin tolerance test was performed by intraperitoneal injection of 0.75 U/kg BW of human regular insulin (Novo Nordisk, Bagsvaerd, Denmark) after 4-h fasting at 18-week of age. Blood glucose levels were monitored at 0, 15, 30, 60, 90, 120 min after insulin injection.

### Measurement of biochemical markers

Blood samples were collected from tail vein. Blood glucose levels were measured using a glucometer (Glutest Mint; Sanwa Kagaku Kenkyusho Co., Ltd, Japan). Plasma total cholesterol and triglyceride levels were measured enzymatically using the Wako LabAssay, L type Wako (Wako Pure Chemical Industries, Japan). Urine was collected using metabolic cage at 18 weeks of age, and urinary 8-OHdG levels were measured using ELISA kit (Japan Institute for the Control of Aging, NIKKEN SEIL Co, Ltd, Japan).

### RNA isolation and real time RT-PCR

Total RNA extraction was performed using a RNeasy lipid tissue mini kit (QIAGEN, Valencia, CA) according to the manufacturers’ instructions. cDNA was produced from mRNA using TaqMan reverse transcription reagents (Applied Biosystems, Foster City, CA). Quantitative RT-PCR was conducted using a Step One Plus Real-Time PCR system (Applied Biosystems). To quantify gene expression, the 2^−ΔCT^ was calculated using β-actin as an internal control. Primer sequences used for real time PCR are presented in Table 1.

**Table 1:** Primer sequences for mouse real-time PCR forward and reverse primers.

Genes	Forward	Reverse
β-actin	CGTGAAAAGATGACCCAGATCA	CACAGCCTGGATGGCTACGTA
MCP-1	CTTCCTCCACCACCATGCA	CCAGCCGGCAACTGTGA
IL-1β	TGGTGTGTGACGTTCCCATTA	CGACAGCACGAGGCTTTTTT
IL-6	ACAACCACGGCCTTCCCTA	CATGTGTAATTAAGCCTCCGACTTG
TIMP-1	GCATGGACATTTATTCTCCACTGT	TCTCTAGGAGCCCCGATCTG
PAI-1	TGCATCGCCTGCCATTG	CTTGAGATAGGACAGTGCTTTTTCC
VCAM-1	GATCTCCCCTGAATACAAAACGAT	GCCCGTAGTGCTGCAAGTG
ICAM-1	TCGGAAGGGAGCCAAGTAACT	CGACGCCGCTCAGAAGAA
MMP-2	CCCTCAAGAAGATGCAGAAGTTC	TCTTGGCTTCCGCATGGT
F4/80	TGCATCTAGCAATGGACAGC	GCCTTCTGGATCCATTTGAA
CD68	TTTCTCCAGCTGTTCACCTTGA	CCCGAAGTGTCCCTTGTCA

### Histological and immunohistological analyses

Under anesthesia, PBS was perfused from left ventricle and then mice were killed and heart and aorta were dissected. Sudan IV (Wako: 192-04392) staining was conducted for aortic arch. Adventitial fat tissue was removed and aorta was dissected longitudinally. The image analysis software NIH Image (version 1.61; http://rsbweb.nih.gov/ij/) was used to calculate the ratio of the plaque lesion to the total aortic arch area.

### Cell culture experiments

HASMCs (third-generation cryopreserved; KURABO Industries, Osaka, Japan) were cultured in Humedia SG-2 (KURABO Industries, Osaka, Japan), following the manufacturers’ recommendations, with cells from passages 4–6 used in the experiments. HASMCs were passaged at 70–80% confluence. Lipopolysaccharide (LPS; 100 ng/mL) was used to induce atherosclerotic changes in HASMCs. Prior to LPS treatment, cells were cultured in serum-free HASMC medium for 24 h^25,26^. After that, the cells were cultured in the presence of LPS with and without esaxerenone (10 nM and 100 nM) (3, 4) for 24 h at 37 °C^22,27^. The cells were then collected in Buffer RLT (Qiagen) with 1% β-mercaptoethanol, and the lysates were stored at − 80 °C. Quantitative RT-PCR was performed using the Step One Plus real-time PCR system (Applied Biosystems), as in the in vivo experiments. Primer sequences used for real time PCR are presented in Supplementary Table 3. To quantify gene expression, we calculated 2^−ΔCT^ using β-actin as an internal control, and quantitatively evaluated the expression of *IL-6, IL-1β, VCAM, and ICAM*^28–30^.

### Statistical analysis

All data were analyzed and expressed as the mean ± standard error of the mean. Differences between two groups were tested for statistical significance using Student’s t-test. *p* values less than 0.05 were considered to indicate a statistically significant difference.

## Results

### No significant difference in atherosclerosis factors between control and esaxerenone group

**Fig. 1:**
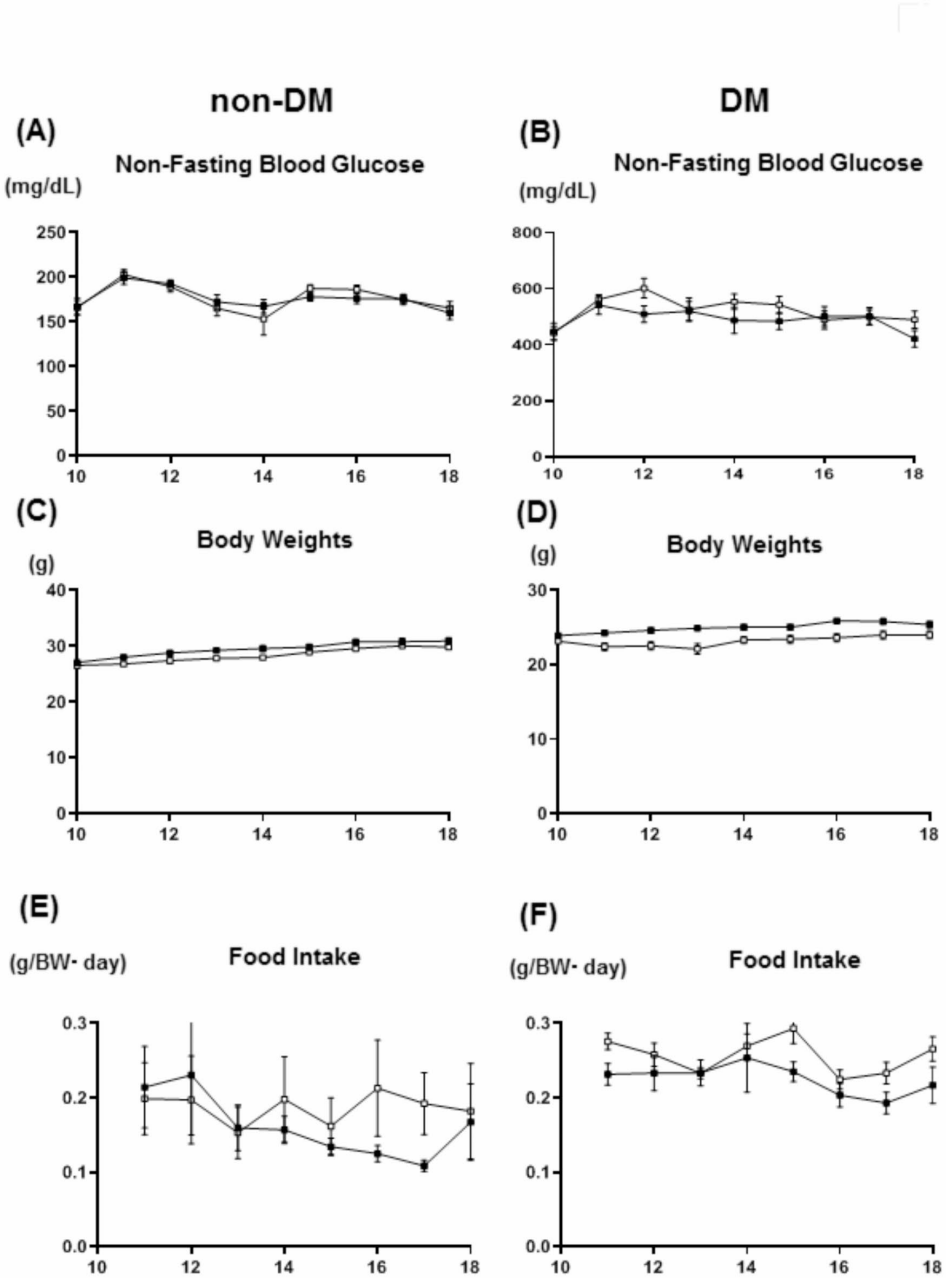
There was no significant difference between esaxerenone and control group in (**A**, **B**) non-fasting blood glucose levels (**A**, **B**), body weights (**C**, **D**), and food intake in non-diabetic and diabetic mice (**E**, **F**) (n = 9–16). Black, control group; white, esaxerenone group.

It is well known that blood glucose, lipid and blood pressure control greatly affect the progression of atherosclerosis. Therefore, we measured blood glucose levels, lipid levels, body weights and blood pressure. In non-diabetic mice, there was no significant difference in non-fasting blood glucose levels between control and esaxerenone group (Fig. 1A). Body weights and food intake were also not different between control and esaxerenone group (Fig. 1C,E). In diabetic mice, there was also no significant difference in non-fasting blood glucose levels, body weights and food intake between the two groups (Fig. 1B,D,F). In fasting conditions, there was also no difference in blood glucose levels, body weights, and lipid levels such as triglyceride and total cholesterol between control and esaxerenone group in non-diabetic and diabetic mice (Tables 2, 3). In addition, OGTT and ITT were performed in the control group, the esaxerenone group, and the spironolactone group, but no significant differences in glucose tolerance were observed among the 3 groups (Supplementary Fig. 1). Esaxerenone is used as an anti-hypertensive drug in clinical practice. However, esaxerenone did not lower blood pressure and heart rate in both non-diabetic and diabetic mice in this strain (Tables 2, 3). Furthermore, esaxerenone did not affect body composition such as organ weights in both non-diabetic and diabetic models (Supplementary Table 1, 2). Therefore, in this study, treatment with esaxerenone did not change any factors that can influence the progression of atherosclerosis. There were no significant differences in non-fasting blood glucose levels, body weights, food intake, blood pressure and heart rate between the spironolactone and control group.

**Table 2:** Biochemical data, body weights, and blood pressure in non-diabetic mice.

	Before treatment (10 weeks)		After treatment (18 weeks)		*p* value
	Control	Esaxerenone	Control	Esaxerenone	
Fasting blood glucose (mg/dl)	62.7 ± 2.7	65.2 ± 3.0	103.5 ± 13.1	87.3 ± 6.8	n.s
Total cholesterol (mg/dl)	Not measured	Not measured	142.2 ± 12.5	152.0 ± 14.4	n.s
Triglyceride (mg/dl)	Not measured	Not measured	94.2 ± 9.6	93.1 ± 12.7	n.s
Fasting body weights (g)	22.9 ± 0.7	22.8 ± 1.1	26.3 ± 0.6	26.5 ± 1.3	n.s
Systolic BP (mmHg)	103.8 ± 2.6	98.1 ± 3.8	96.1 ± 2.5	98.8 ± 4.5	n.s
Diastolic BP (mmHg)	55.5 ± 2.6	51.1 ± 2.5	50.0 ± 4.3	41.1 ± 5.3	n.s
Heart rate (beats/min)	703.8 ± 10.3	662.9 ± 18.3	711.1 ± 12.7	694.0 ± 13.7	n.s

*p* value between with and without esaxerenone treatment at 18 weeks. n.s.: not significant.

**Table 3:** Biochemical data, body weights, and blood pressure in diabetic mice.

	Before treatment (10 weeks)		After treatment (18 weeks)		*p* value
	control	Esaxerenone	control	Esaxerenone	
Fasting blood glucose (mg/dl)	120.6 ± 13.5	160.3 ± 12.7	154.1 ± 20.5	138.1 ± 14.8	n.s
Total cholesterol (mg/dl)	Not measured	Not measured	176.8 ± 19.0	174.3 ± 41.0	n.s
Triglyceride (mg/dl)	Not measured	Not measured	123.5 ± 12.7	153.6 ± 11.4	n.s
Fasting body weights (g)	21.1 ± 0.55	19.6 ± 1.38	23.6 ± 0.82	26.1 ± 0.72	n.s
Systolic BP (mmHg)	97.1 ± 2.5	104.0 ± 3.0	105.4 ± 3.2	104.1 ± 8.3	n.s
Diastolic BP (mmHg)	47.6 ± 4.2	50.6 ± 3.5	49.5 ± 5.8	50.4 ± 4.8	n.s
Heart rate (beats/min)	660.9 ± 17.2	648.2 ± 14.5	711.1 ± 12.7	694 ± 13.7	n.s

*p* value between with and without esaxerenone treatment at 18 weeks. n.s.: not significant;

### Plaque formation in the aortic arch was significantly reduced in esaxerenone group in diabetic mice

**Fig. 2:**
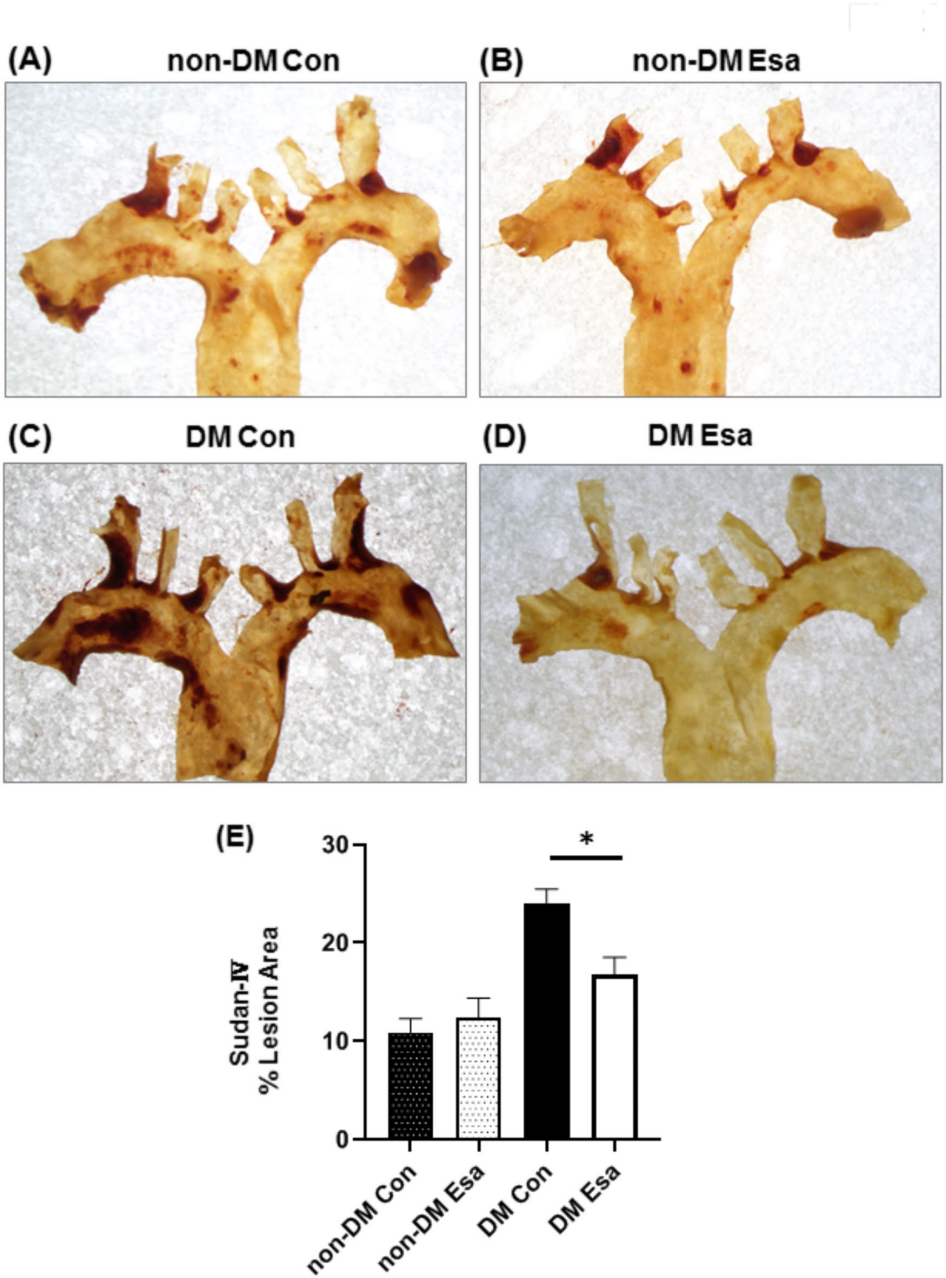
Atherosclerotic lesion in aortic arch in non-DM Control (**A**), non-DM Esaxerenone (**B**), DM Control (**C**) and DM Esaxerenone (**D**). (**E**) Esaxerenone treatment significantly reduced plaque area compared to control group in diabetic mice. n = 8. *: *p* < 0.05.

Next, to investigate the effect of esaxerenone on the progression of atherosclerosis, plaque formation in the aortic arch was evaluated. The area of plaque stained with Sudan IV was measured and compared between with and without esaxerenone treatment. In the non-diabetic mice, there was no significant difference between control and esaxerenone group (Fig. 2A,B,E). However, esaxerenone treatment significantly reduced plaque area compared to control group in diabetic mice (Fig. 2C–E). In the spironolactone-treated group, there was no significant decrease in plaque area in either diabetic or non-diabetic mice (Supplementary Fig. 1).

### Esaxerenone treatment reduced oxidative stress and inflammatory cytokines in diabetic mice

**Fig. 3:**
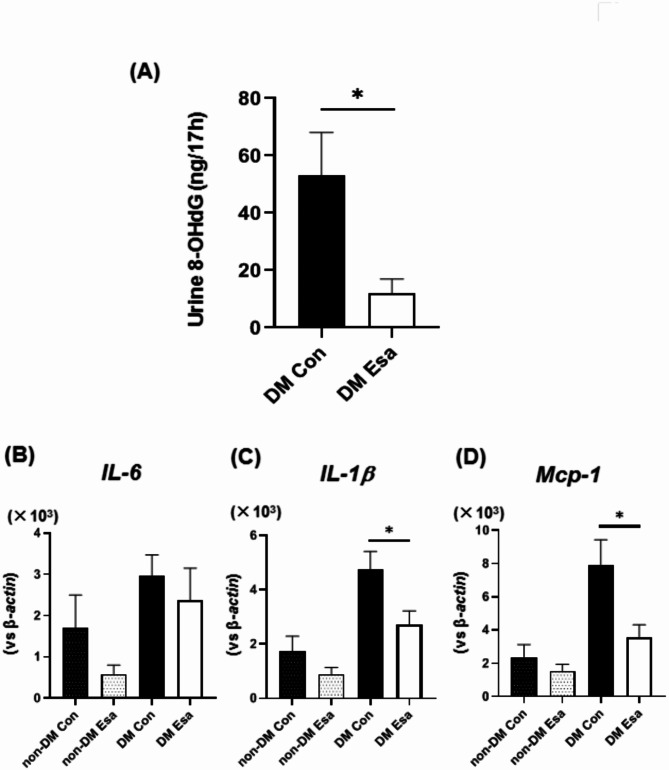
(**A**) In esaxerenone group, urinary 8-OHdG levels were significantly lower compared to control in diabetic mice. n = 8. (**B**–**D**) mRNA expression levels of inflammatory markers in the abdominal aorta were lower in esaxerenone group compared to control in diabetic mice. n = 12. *: *p* < 0.05.

Next, we examined the possible effects of esaxerenone on the progression of atherosclerosis. It is known that activation of the RAS stimulates NADPH oxidase and increase reactive oxygen species (ROS)^31^ . Therefore, we evaluated urinary 8-OHdG levels as oxidative stress marker. Urinary 8-OHdG levels were significantly lower in esaxerenone group compared to control group in diabetic mice (Fig. 3A). Expression levels of inflammatory cytokines such as *Il-6*, *Il-1β* and *Mcp-1* were higher in diabetic mice compared to non-diabetic mice. In non-diabetic mice, expression levels of *Il-6*, *Il-1β* and *Mcp-1* tended to be lower in esaxerenone group, although it did not reach a statistical significance. In diabetic mice, however, expression levels of *Il-1β* and *Mcp-1* were significantly lower in esaxerenone group compared to control (Fig. 3B–D). Taken together, specific blocking of mineralocorticoid signaling with esaxerenone reduced ROS production and decreased expression levels of inflammatory cytokines in abdominal aorta under diabetic conditions.

### Esaxerenone reduced expression levels of factors associated with plaque formation and stability in the aorta of diabetic mice

**Fig. 4:**
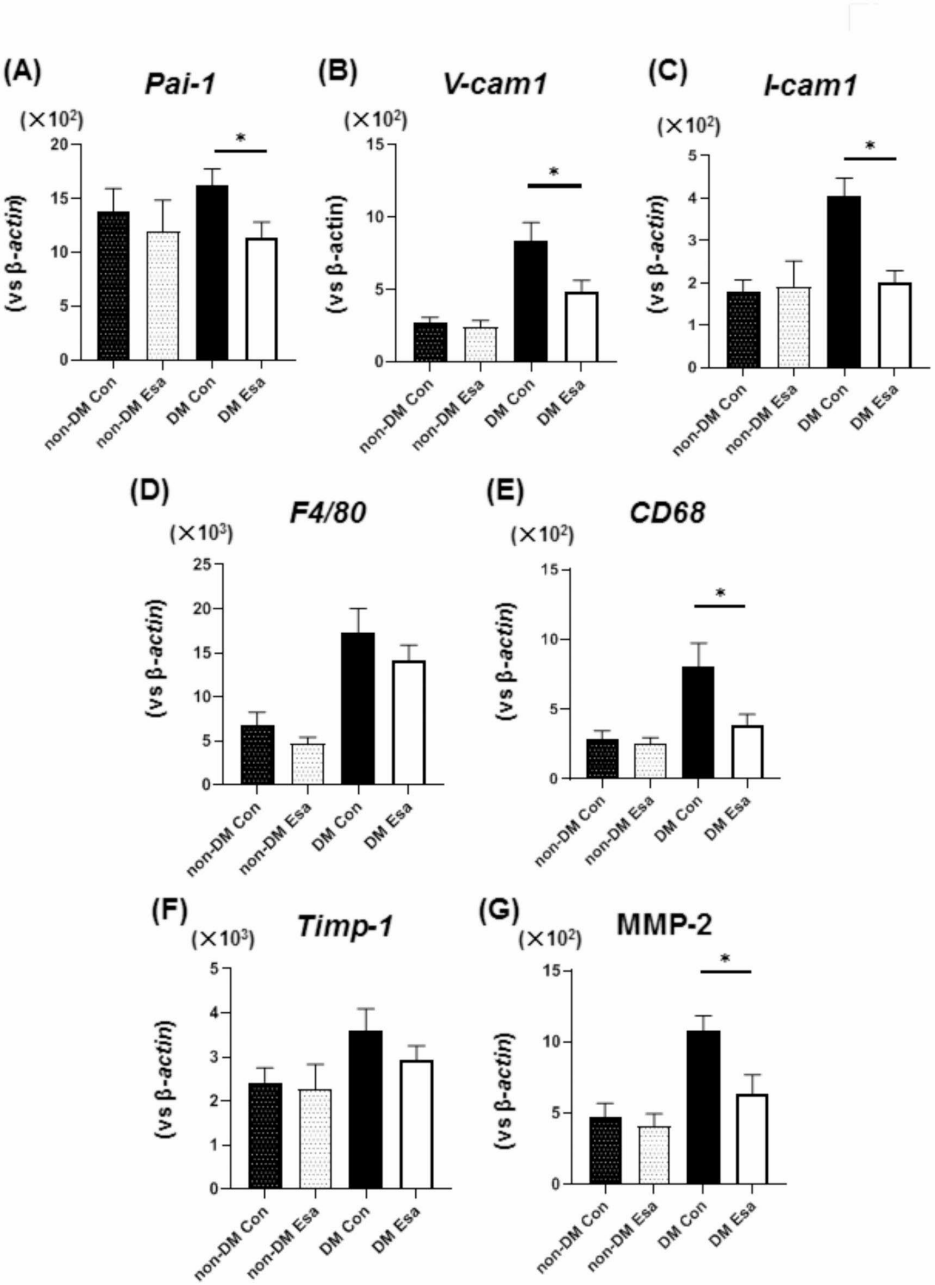
(**A**–**C**) mRNA expression levels related to coagulation such as PAI-1, cell adhesion molecules such as V-CAM and I-CAM were significantly lower in esaxerenone group compared to control in diabetic mice. (**D**) Expression level of a macrophage marker F4/80 was not different between control and esaxerenone group. (**E**) Expression level of CD68 was significantly lower in esaxerenone group compared to control in diabetic mice. (**F**) Expression level of a plaque stability marker TIMP-1 was not different between the control and esaxerenone group. (**G**) Expression levels MMP-2 was significantly lower in esaxerenone group compared to control in diabetic mice. n = 12. *: *p* < 0.05.

Finally, to examine other factors associated with atherosclerosis, we evaluated expression levels of cell adhesion, macrophage and plaque stability markers in the abdominal aorta. In non-diabetic mice, there were no differences in expression levels of cell adhesion, macrophage and plaque stability markers. Expression levels related with thrombus formation or cell adhesion such as *Pai-1*, *Vcam-1*, *Icam-1* were significantly lower in esaxerenone group in diabetic mice (Fig. 4A–C). There was no difference in macrophage marker such as *F4/80*, but expression levels of *Cd68* were significantly lower in esaxerenone group compared to control in diabetic mice (Fig. 4D,E). Regarding plaque stability, there was no difference in expression levels of Timp-1 (Fig. 4F). Mmp-2 expression level was significantly lower in esaxerenone group in diabetic mice (Fig. 4G).

### Esaxerenone has anti-inflammatory effects in human smooth muscle cells

**Fig. 5:**
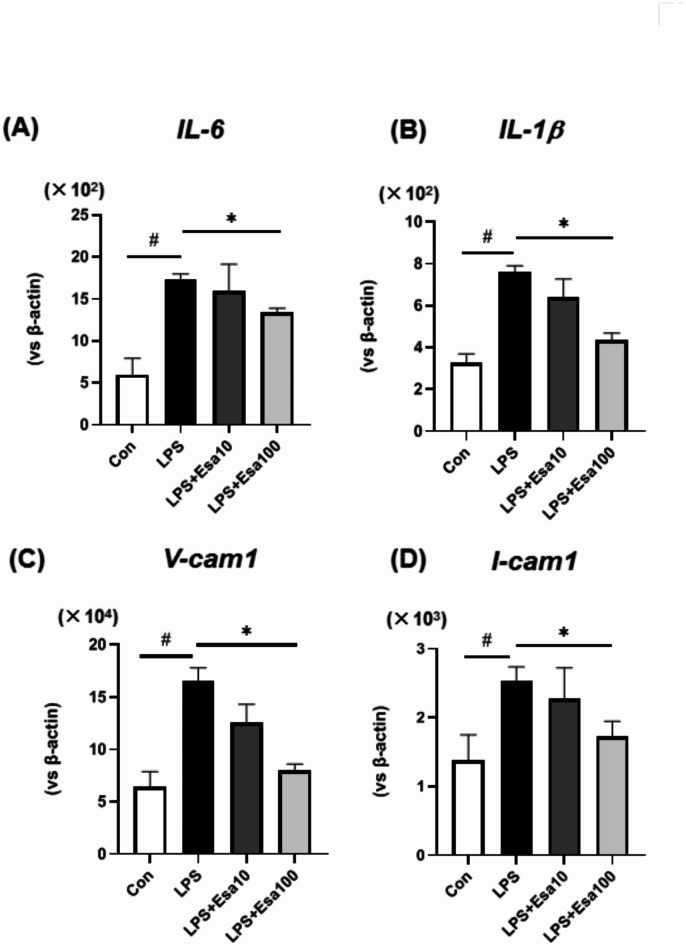
(**A**, **B**) The mRNA expression levels of inflammatory markers in human aortic smooth muscle cells (HASMCs) were significantly lower in the group treated with 100 nM of esaxerenone than in the group treated with LPS alone. (**C**, **D**) The mRNA expression levels of V-CAM and I-CAM, which are related to cell adhesion molecules, were also significantly lower in the group treated with 100 nM of esaxerenone than in the group treated with LPS alone. Con: control. LPS: stimulated with 100 ng/mL of LPS. LPS + Esa10: 10 nM of esaxerenone was administered together with LPS stimulation. LPS + E100: 100 nM of esaxerenone was administered together with LPS stimulation. n = 3. #: *p* < 0.05, Con versus LPS. *: *p* < 0.05, LPS versus LPS + Esa100.

In order to clarify the molecular mechanism of the anti-atherosclerotic effect of esaxerenone, we conducted cell culture experiments. In HASMC cells which were stimulated with 100 ng/mL of LPS, treatment with 100 nM of esaxerenone significantly reduced the expression levels of IL-6, IL-1β, VCAM, and ICAM compared to untreated cells (Fig. 5). The same evaluation was also conducted in various arterial cell types, including human aortic endothelial cells, as well as macrophage strains such as THP-1. However, no significant differences were observed between the groups with and without esaxerenone treatment (data not shown).

## Discussion

In this study, we demonstrated that specific blocking of mineralocorticoid signaling with esaxerenone exerted favorable anti-atherosclerotic effects in the aorta of STZ-induced hyperglycemic ApoE KO mice. Furthermore, interestingly, such anti-atherosclerotic effects were obtained without influencing blood pressure and glycolipid metabolism. Other MR blockers, such as spironolactone^32^ and eplerenone^33,34^ showed anti-atherosclerotic effects, but both drugs decreased blood pressure and improved lipid profile both of which are well known to reduce the progression of atherosclerosis. Therefore, while mineralocorticoid signaling has been drawing much attention recently, this is the first report which clearly demonstrates that blocking of mineralocorticoid signaling exerts favorable anti-atherosclerotic effects, independently of blood pressure and glycolipid metabolism.

In the FIGARO-DKD study, finerenone significantly reduced the risk of a composite vascular endpoint by 13% in patients with chronic kidney disease and type 2 diabetes^20^, but basic studies have only examined cardioprotection and renoprotection^18,19^. There is no report examining its possible effects on atherosclerosis. It is thought that esaxerenone suppresses the progression of atherosclerosis by suppressing mineralocorticoid signaling and reducing oxidative stress and inflammation. In this study, urinary 8-OHdG, an oxidative stress marker, was lower in esaxerenone group compared to control group. Expression levels of inflammatory markers such as *Il-1β* and *Mcp-1* were significantly lower in esaxerenone group in diabetic mice. Furthermore, expression levels of *Pai-1*, *Cd68*, *Vcam-1* and *Icam-1* may have decreased due to the improvement of inflammation. It has been reported that MR-mediated NADPH oxidase and Rac1 activation induces the production of ROS and are involved in vascular injury^35,36^. We think that esaxerenone blocked MR and decreased ROS production. It is also known that MR activation promotes leukocyte adhesion through increased *Icam-1* expression^37^ and production of inflammatory cytokines and chemokines such as *Il-1β*, *Mcp-1*, and* Pai-1*^38^ In this study, inflammatory cytokines and cell adhesion factors were increased under diabetic conditions, and these were significantly improved by esaxerenone treatment.

On the other hand, there were no significant differences in blood pressure, lipid profile, and blood glucose levels between esaxerenone and control group. Esaxerenone is a drug used clinically for hypertension, but the selected dosage did not affect blood pressure as previously reported^39,40^. There was no difference in blood pressure in these reports, but esaxerenone showed beneficial effects on vascular dysfunction. A similar study was also conducted with spironolactone, but no significant reduction in plaque area was observed.

In addition, in a study in which LPS stimulation was performed in HASMCs, a significant decrease in inflammatory response was observed with the administration of esaxerenone. It was reported that aldosterone induces inflammation in rat vascular smooth muscle cells via the mineralocorticoid receptor, reactive oxygen species, the MAPK pathway and the NF-κB pathway^41^, and it has also been shown that the mineralocorticoid receptor in vascular smooth muscle cells is extremely important for arteriosclerosis caused by aldosterone and salt using VSCM-specific MR knockout mice^42^. It is thought that aldosterone may accelerate the development of atherosclerosis by increasing the cellular responsiveness to IGF-1, thereby promoting the proliferation, migration and protein synthesis of VSMCs stimulated by IGF-1^43^. Furthermore, aldosterone is involved in the development of aortic injury by activating MR-induced pyroptosis in VSMCs, and there are reports that esaxerenone protects the aorta by inhibiting mitochondrial oxidative damage and VSMC pyroptosis^44^. There is a report that esaxerenone suppresses cardiac dysfunction, fibrosis, and inflammation independent of genomic regulation in a myocardial infarction model mouse^45^. Based on the above, it is thought that the main factor in the anti-arteriosclerotic effect of esaxerenone is its direct anti-inflammatory effect on VSMCs. In addition, we think the possibility that esaxerenone directly exerts favorable anti-atherosclerotic effects by reducing oxidative stress and inflammation.

There is limitation in this study. First, there was significant improvement in plaque area and mRNA levels in diabetic mice, however no significant improvement was observed in non-diabetic mice. In non-diabetic mice, plaque formation was significantly less compared to diabetic mice, which may explain the lack of significant differences. Second, although there was no significant difference in expression levels of mRNA, inflammatory cytokines showed a decreasing trend. Therefore, if the intervention period is extended, significant differences may occur even under non-diabetic conditions.

## Conclusion

In conclusion, specific blocking of mineralocorticoid signaling with esaxerenone exerts favorable effects on the development of plaque formation and progression of atherosclerosis, which was independent of blood pressure and glycolipid metabolism. To the best of our knowledge, this is the first report showing that MR blocking per se exerts favorable anti-atherosclerotic effects presumably due to reduction of oxidative stress and/or inflammation, independently of blood pressure and glycolipid metabolism. Therefore, we think that the data obtained in this study would be informative and useful in atherosclerosis research area as well as from the clinical point of view.

## Electronic supplementary material

Below is the link to the electronic supplementary material.

Supplementary Material 1

Supplementary Material 2

## Data Availability

The datasets generated and/or analyzed during the current study are available from the corresponding author upon reasonable request.

## Abbreviations

ApoE: Apolipoprotein E
STZ: Streptozotocin
TG: Triglyceride
LDL-C: Low density lipoprotein cholesterol
HDL-C: High density lipoprotein cholesterol
ROS: Reactive oxygen species
8-OHdG: 8-Hydroxy-2’-deoxyguanosine
*Il-1β*: Interleukin 1 β
*Mcp-1*: Monocyte chemotactic protein-1
*iNos*: Inducible nitric oxide synthase
*Vcam-1*: Vascular cell adhesion molecule
*Icam-1*: Intracellular adhesion molecule
*Timp-1*: Tissue inhibitor metalloproteinase 1
*Mmp-2*: Matrix metalloproteinase 2
