# Supplementary material for: Blocking mineralocorticoid signaling with esaxerenone reduces atherosclerosis in hyperglycemic ApoE KO mice without affecting blood pressure and glycolipid metabolism
Source: Sci Rep. 2025 Mar 29;15:10887. doi: 10.1038/s41598-025-95324-z (PMC11954868; doi:10.1038/s41598-025-95324-z)
Supplement: Supplementary file 1 — Supplementary Material 1 [file 41598_2025_95324_MOESM1_ESM.pptx]

## Slide 1
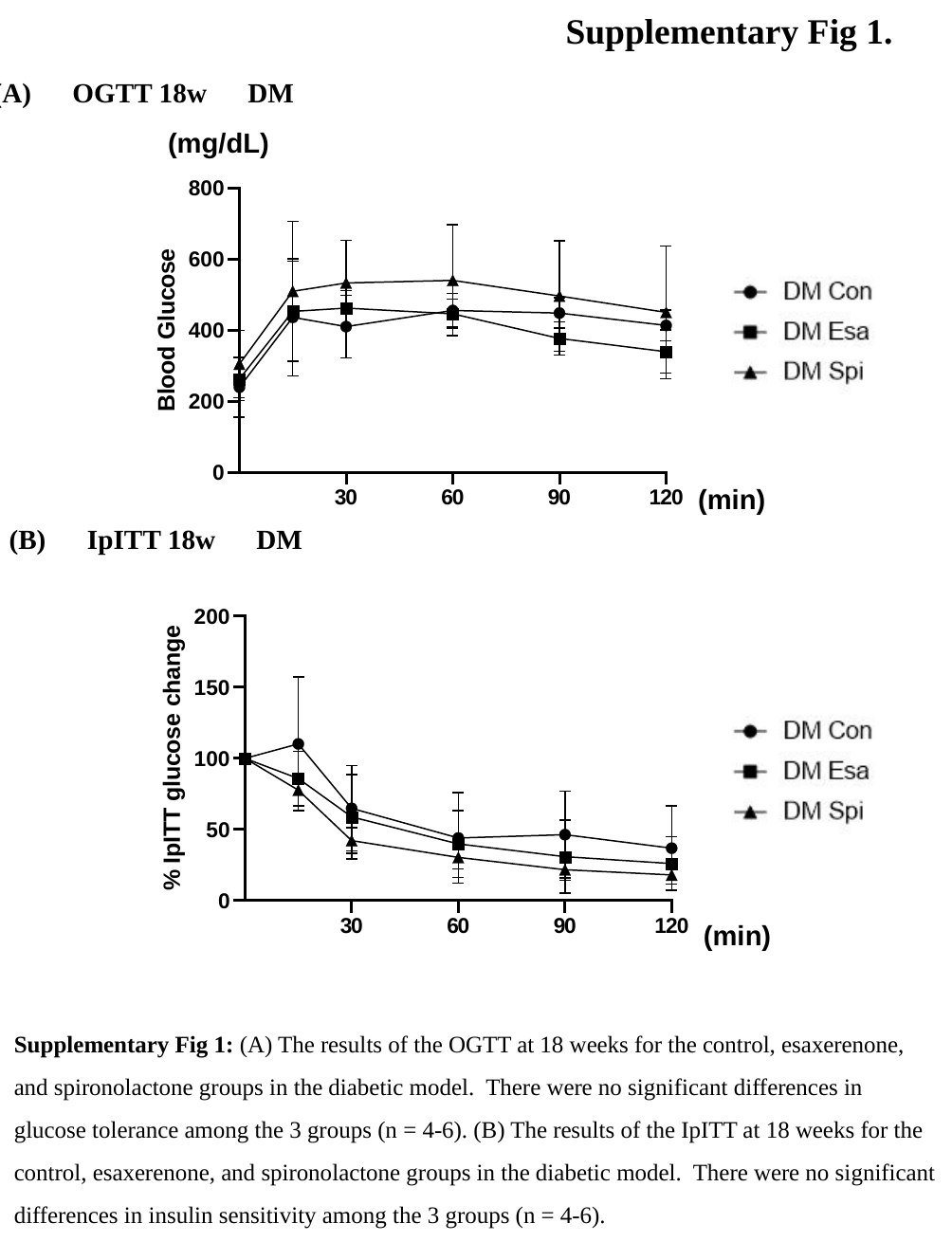

Supplementary Fig 1.
(A)　OGTT 18w　DM
(mg/dL)
(min)
(B)　IpITT 18w　DM
(min)
Supplementary Fig 1: (A) The results of the OGTT at 18 weeks for the control, esaxerenone, and spironolactone groups in the diabetic model. There were no significant differences in glucose tolerance among the 3 groups (n = 4-6). (B) The results of the IpITT at 18 weeks for the control, esaxerenone, and spironolactone groups in the diabetic model. There were no significant differences in insulin sensitivity among the 3 groups (n = 4-6).

## Slide 2
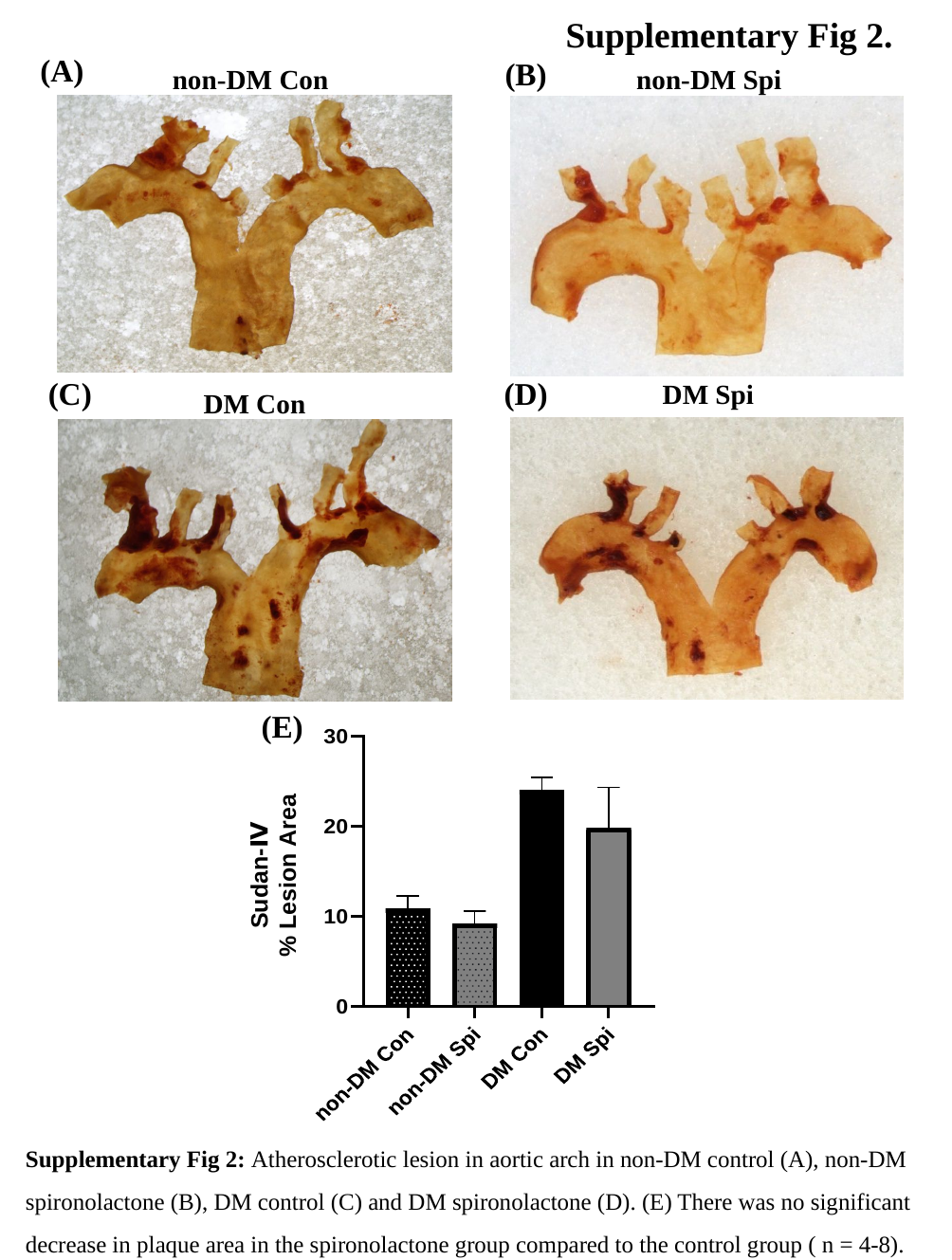

Supplementary Fig 2.
(A)
(B)
non-DM Spi
non-DM Con
(C)
(D)
DM Spi
DM Con
(E)
Sudan-Ⅳ
% Lesion Area
Supplementary Fig 2: Atherosclerotic lesion in aortic arch in non-DM control (A), non-DM spironolactone (B), DM control (C) and DM spironolactone (D). (E) There was no significant decrease in plaque area in the spironolactone group compared to the control group ( n = 4-8).
