# Supplementary material for: Blocking mineralocorticoid signaling with esaxerenone reduces atherosclerosis in hyperglycemic ApoE KO mice without affecting blood pressure and glycolipid metabolism
Source: Sci Rep. 2025 Mar 29;15:10887. doi: 10.1038/s41598-025-95324-z (PMC11954868; doi:10.1038/s41598-025-95324-z)
Supplement: Supplementary file 2 — Supplementary Material 2 [file 41598_2025_95324_MOESM2_ESM.docx]

Supplementary Table 1：Organ weights in non-diabetic mice

|  | After treatment (18 weeks) | |  |
| --- | --- | --- | --- |
|  | Control | Esaxerenone | *p* value |
| Pancreas (g) | 0.228 ± 0.03 | 0.260 ± 0.03 | n.s. |
| Heart (g) | 0.118 ± 0.004 | 0.125 ± 0.01 | n.s. |
| Kidney(g) | 0.360 ± 0.02 | 0.369 ± 0.01 | n.s. |
| Liver (g) | 1.19 ± 0.05 | 1.207 ± 0.07 | n.s. |
| Muscle (g) | 0.350 ± 0.02 | 0.297 ± 0.03 | n.s. |
| White adipo tissue (g) | 0.304 ± 0.05 | 0.67 ± 0.30 | n.s. |
| Brown adipo tissue (g) | 0.136 ± 0.02 | 0.147 ± 0.01 | n.s. |

*p* value between with and without esaxerenone treatment at 18 weeks. n.s.: not significant.

(n=10-12)

Supplementary Table 2：Organ weights in diabetic mice

|  | After treatment (18 weeks) | |  |
| --- | --- | --- | --- |
|  | Control | Esaxerenone | *p* value |
| Pancreas (g) | 0.230 ± 0.01 | 0.216 ± 0.03 | n.s. |
| Heart (g) | 0.097 ± 0.003 | 0.090 ± 0.004 | n.s. |
| Kidney(g) | 0.321 ± 0.01 | 0.313 ± 0.01 | n.s. |
| Liver (g) | 1.05 ± 0.07 | 1.11 ± 0.03 | n.s. |
| Muscle (g) | 0.262 ± 0.02 | 0.287 ± 0.02 | n.s. |
| White adipose tissue (g) | N/A | N/A | N/A |
| Brown adipose tissue (g) | 0.115 ± 0.02 | 0.115 ± 0.02 | n.s. |

*p* value between with and without esaxerenone treatment at 18 weeks. n.s.: not significant;

N/A: not applicable

(n=10-12)

Supplementary Table 3：Primer sequences for human real-time PCR forward and reverse primers

| Genes | Forward | Reverse |
| --- | --- | --- |
| β-actin | CACCATTGGCAATGAGCGGTTC | AGGTCTTTGCGGATGTCCACGT |
| IL-1β | CCACAGACCTTCCAGGAGAATG | GTGCAGTTCAGTGATCGTACAGG |
| IL-6 | AGACAGCCACTCACCTCTTCAG | TTCTGCCAGTGCCTCTTTGCTG |
| VCAM-1 | CCCTGGAAACCAAGAGTTTGGA | TCTTGCAGCTTTGTGGATGGA |
| ICAM-1 | AACTGACACCTTTGTTAGCCACCTC | CCCAGTGAAATGCAAACAGGAC |
